# Evolutionary integration and modularity in the diversity of the suckermouth armoured catfishes

**DOI:** 10.1098/rsos.220713

**Published:** 2022-11-23

**Authors:** Corinthia R. Black, Jonathan W. Armbruster

**Affiliations:** Department of Biological Sciences, Auburn University, Auburn, AL, USA

**Keywords:** neotropical, geometric morphometrics, shape, morphological evolution, phylogenetic comparative methods, Loricariidae

## Abstract

The evolution of morphological diversity has held a long-standing fascination among scientists. In particular, do bodies evolve as single, integrated units or do different body parts evolve semi-independently (modules)? Suckermouth armoured catfishes (Loricariidae) have a morphology that lends nicely to evolutionary modularity and integration studies. In addition to a ventrally facing oral jaw that directly contacts surfaces, the neurocranium and pectoral girdle are fused, which limits movement of the anterior part of the body. Functional constraints suggest it is likely the head and post-cranial body act as separate modules that can evolve independently. If true, one would expect to see a two- or three-module system where the head and post-cranial body are morphologically distinct. To test this hypothesis, we quantified shape using geometric morphometric analysis and assessed the degree of modularity across functionally important regions. We found the armoured catfish body is highly modularized, with varying degrees of integration between each module. Within subfamilies, there are different patterns of evolutionary modularity and integration, suggesting that the various patterns may have driven diversification along a single trajectory in each subfamily. This study suggests the evolution of armoured catfish diversification is complex, with morphological evolution influenced by interactions within and between modules.

## 1. Introduction

The evolution of morphological diversity is often influenced by extrinsic factors, like functional constraints and ecological interactions [1–3]. However, intrinsic properties, such as modularity and integration, have been shown to enhance or constrain the evolution of form [4–6]. Modularity and integration are closely related concepts that investigate how different units within an organism correlate to one another [7]. Although these concepts are closely related, modularity refers to how parts of the body change as independent units, whereas integration describes the coordinated interactions between parts of the body where changes in one area effect the changes in another [8]. Modularity and integration can be used to explain coordination among structures (morphological integration), covariation in size or shape within a population (phenotypic integration), and coordination of structures to perform functions (functional coordination/coupling) [7,9–12]. More specifically, evolutionary modularity and integration refer to the correlation in shape across evolutionary history [9,13–15]. Evolutionary modularity has been traditionally hypothesized to accelerate diversification, as independent modules have the ability to evolve separately from one another, while integration is hypothesized to constrain the evolution of form due to the tight interactions of parts that prevent rapid changes. However, recent studies have suggested that modularity and integration is not as simple as previously thought. In a tightly integrated system, changes in an individual trait will probably cause a cascading effect where traits that share common functions, genetics and/or developmental origins will change in response to the original change. These coordinated interactions may cause tightly integrated organisms to be morphologically constrained or diverse [16]. For example, some studies have demonstrated that highly integrated systems have been linked to an increase in diversity along a single trajectory which may be influenced by coordinated morphological changes under selected pressures [16,17].

Additionally, integration and modularity are not all or nothing concepts, but more a matter of degree. For example, Klingenberg *et al*. [18] discovered that the lower jaw of the mouse skull has distinct developmental modules; however, they are not completely independent of each other. Although the alveolar region is distinct from the ramus, changes in one will affect changes in the other to some degree. This type of relationship has been seen in fishes, where African cichlids, a clade known for rapid radiation, shows integration between the oral and pharyngeal jaws. Integration between modules was previously hypothesized to limit the evolvability of morphology but seems to work as a feature to promote radiations in cichlids [19]. Integration may suggest that changes in function, such as feeding method, may have broad effects across the morphology of the organism. This subsequently may allow for broader ecological change stemming from a simple change in one module. Such modularity and integration would suggest that convergence in one module may lead to corresponding similar changes throughout the integrated modules.

Geography and subsequent changes of said geography can also attribute to diversification within animals. In South America, highland areas include the very old Brazilian and Guiana Shields (part of the Amazon Craton) and the much younger Andes mountains, which are separated from one another by lowlands that have been occasionally flooded by marine incursions. With limited dispersal for upland fauna between the Brazilian and Guiana Shields and between the shields and Andes, the interplay of modularity and integration sets up a system whereby convergence in body form is likely to occur.

The suckermouth armoured catfishes (Loricariidae) are a group of neotropical fishes that are incredibly diverse in morphology and ecology. Consisting of over 1035 species in 100 genera, loricariids are considered the most species-rich family in the order of Siluriformes [20]. Many new species are described each year, making the loricariid catfishes a dynamic and growing group of freshwater fishes [21,22]. The family is monophyletic and united by three traits, ossified dermal plates that cover the body, integumentary teeth known as odontodes on bony plates and fin spines, and a ventral oral disc used in feeding and to adhere to objects in their habitats [23–26].

The unique morphology and evolutionary history of armoured catfishes lend nicely to evolutionary modularity and integration studies. Loricariids have a highly mobile ventrally facing oral jaw that directly contacts surfaces, whereas the neurocranium and pectoral girdle are fused limiting movement of the anterior part of the body. The lack of movement due to this fusion in addition to a specialized mode of feeding suggests the mouth and neurocranium/pectoral girdle may form independent modules.

Schaefer & Lauder [27,28] proposed a set of significant decouplings (as well as new biomechanical couples) in loricariid catfishes. Although there had been some changes early in loricarioid phylogeny, loricariids plus astroblepids share a number of changes and losses to biomechanical couples. These changes are hypothesized to have functionally decoupled the jaws; a new division of the adductor mandibulae operates the premaxillae, and the left and right lower jaws are decoupled from one another and can move independently. Furthermore, the jaws lost a coupling of the opercular complex (interopercular–mandibular ligament), although that couple appears to have re-evolved multiple times [29]. In addition to a ventrally facing oral jaw that directly attaches to surfaces, the neurocranium and pectoral girdle are fused, which limits movement of the anterior part of the body.

With the jaws’ ability to move independently of the skull and the limitations to movement within the neurocranium and pectoral girdle, we hypothesize that changes in jaw, neurocranium and postcranial morphology could act as separate modules that could evolve somewhat independently of one another. This gives the possibility of swapping jaw modules without considerable changes to much of the rest of the anatomy. However, integration may still play a role, as there are limitations to form. For example, a long dentary bone in a narrow head would not logically be possible. This suggests that changes in jaw morphology could lead to a series of changes elsewhere in the body. To test for the degree of modularity and integration across functionally important regions within the armoured catfishes, we quantified shape using geometric morphometric analysis and tested different modular hypotheses.

## 2. Material and methods

### 2.1. Data collection

**Figure 1.**
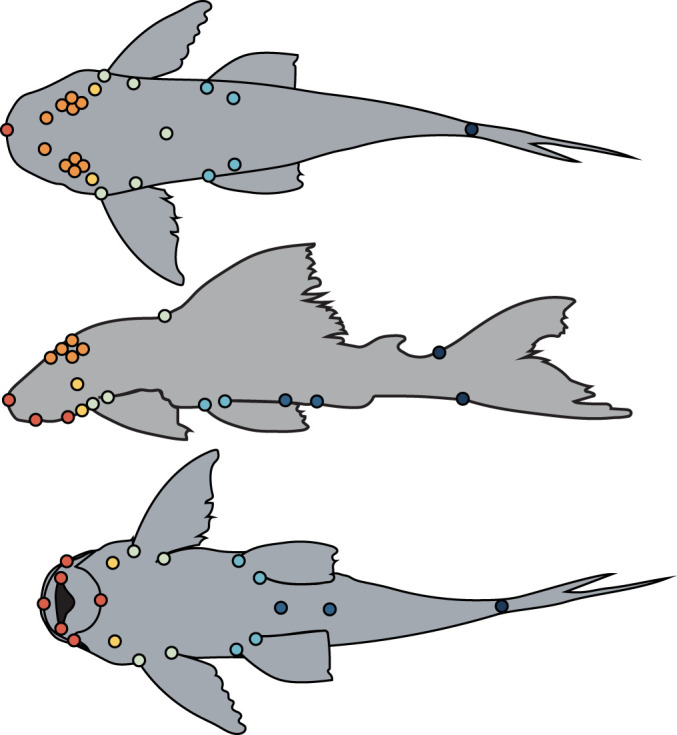
Landmark scheme and modules on a representative catfish in dorsal, left lateral and ventral views. Colours correspond to body regions of distinct modules: red = mouth (tip of snout, left and right lateral joints of mouth and most lateral and posterior parts of the oral disc), orange = neurocranium (left and right naris, anterior, dorsal, posterior and ventral points of the eyes), yellow = opercula (most ventral and dorsal slits of opercula), light green = pectoral and dorsal fins (origin and insertion of the pectoral fins and origin of dorsal fin), light blue = pelvic fins (origin and insertion of the pelvic fins), dark blue = anal area (cloaca and anal-fin origin), dark purple = caudal peduncle (dorsal and ventral points of the caudal peduncle).

A total of 209 adult specimens representing 71 species within the Loricariidae were photographed from various fish collections. Four subfamilies were represented by the following number of species; Hypoptopomatinae *n* = 6 (255 total species), Hypostominae *n* = 50 (498 total species), Lithogeninae *n* = 1 (3 total species) and Loricariinae *n* = 13 (258 total species) [20]. Although this study represents a small portion of all loricariid specimens (approx. 7% of the family), the species in this study are broadly placed across known phylogenetic hypotheses to represent major morphologies of the group and were based on availability of specimens. All specimens are obtained from museum collections (table 1) and therefore do not require ethical approval and collection permits. Thirty-three landmarks that capture overall body shape were modified from Armbruster [30] (figure 1). The landmarks were reconstructed into a three-dimensional space using stereo camera reconstruction in the R package StereoMorph for three to five individuals per species (table 1) [31]. Two cameras (Nikon D90 DSLR attached to a copy stand and a Canon Rebel XSi DSLR attached to a tripod) were positioned at an approximately 35-degree angle from one another and calibrated in space using an 8 × 6, 180-pixel chequerboard. To avoid movement of the camera positions, photos were taken using a wireless remote, and autofocus was turned off for the session. Specimens were held in place using moulding clay to avoid movement of the specimen and align the specimen properly. Each specimen was photographed in two aspects, a dorsal and ventral view, to capture the maximum shape variation with landmarks.

**Table 1.** Specimens used in this study.

taxon	abbr. in figure	catalogue number	in Roxo *et al*. [22] (congener)	in Lujan *et al*. [21] (congener)
Loricariidae: Hypoptopomatinae				
*Corumbataia tocantinensis*	*C. toc*	AUM45418, AUM45418, AUM45418, AUM45418	yes (*Corumbataia cuestae* 17210)	yes (*Corumbataia cuestae*)
*Hypoptopoma gulare*	*H. gul*	AUM66085	yes (*Hypoptopoma psilogaster* 22980)	yes (*Hypoptopoma spectabile*)
*Hypoptopoma thoracatum*	*H. tho*	AUM47901, AUM47901	yes	no
*Otocinclus vestitus*	*O. ves*	AUM22715	yes (*Otocinclus vittatus* 26232)	yes (*Otocinclus vittatus*)
*Oxyropsis acutirostra*	*O. acu*	AUM56739	no	yes (*Oxyropsis ephippia*)
*Parotocinclus eppleyi*	*P. epp*	AUM56697	yes (*Parotocinclus* cf. *bahiensis* 34692)	yes (*Parotocinclus bidentatus*)
Loricariidae: Hypostominae				
*Ancistrus bufonius*	*A. buf*	AUM46276	no	no
*Ancistrus chagresi*	*A. cha*	AUM32114	no	no
*Ancistrus damasceni*	*A. dam*	AUM20700	no	no
*Ancistrus leucostictus*	*A. leu*	AUM48762	no	yes
*Ancistrus lithurgicus*	*A. lit*	AUM38182, AUM38821	no	no
*Ancistrus macrophthalmus*	*A. mac*	AUM53526	no	yes
*Ancistrus marcapatae*	*A. mar*	AUM51152	no	no
*Ancistrus nudiceps*	*A. nud*	AUM35624, AUM47720, AUM50295	no	no
*Ancistrus triradiatus*	*A. tri*	AUM22190, AUM22297, AUM54016, AUM54047	no	no
*Aphanotorulus ammophilus*	*A. amm*	AUM27705	yes *(Aphanotorulus unicolor* 19719)	yes
*Baryancistrus beggini*	*B. beg*	AUM54990	yes (*Baryancistrus beggini* 39227)	yes
*Chaetostoma lineopunctatum*	*C. lin*	AUM51201, AUM51341	yes (*Chaetostoma jegui*)	yes
*Cordylancistrus sp.*	*C. sp.*	AUM71150, AUM71168	no	yes (*Cordylancistrus torbesensis*)
*Corymbophanes kaiei*	*C. kai*	AUM62801	no	yes
*Dekeyseria scaphirhynchus*	*D. sca*	AUM44111, AUM54474	no	yes
*Dolichancistrus cobrensis*	*D. cob*	AUM46306	yes (*Dolichancistrus carnegiei* 189598)	yes (*Dolichancistrus carnegiei* 6647)
*Exastilithoxus sp.*	*E. sp.*	AUM54450	yes (*Exastilithoxus hoedemani* 42177)	yes (*Exastilithoxus* nsp Ventuari T09667)
*Hemiancistrus guahiborum*	*H. gua*	AUM53523, AUM53821, AUM56668	yes (*Hemiancistrus punctulatus* 60931)	yes
*Hemiancistrus lujani*	*H. luj*	ANSP162174, AUM43008	yes (*Hemiancistrus fuliginosus* 61299)	no
*Hypancistrus debilittera*	*H. deb*	AUM53528	yes (*Hypancistrus* sp. 61759)	no
*Hypancistrus furunculus*	*H. fur*	AUM54463	no	yes
*Hypancistrus lunaorum*	*H. lun*	AUM42120, AUM44315	no	yes
*Hypostomus niceforoi*	*H. nic*	AUM45519, AUM57497	no	yes
*Hypostomus robinii*	*H. rob*	AUM22244, AUM36436	no	yes
*Isorineloricaria spinosissima*	*I. spi*	AUM4251	no	yes
*Lasiancistrus schomburgkii*	*L. sch*	AUM45574, AUM45627	yes (*Lasiancistrus saetiger* 42517)	yes
*Lasiancistrus tentaculatus*	*L. ten*	AUM39278, AUM53761	no	yes
*Leporacanthicus cf. galaxias*	*L. gal*	AUM54029	yes	yes
*Lithoxus lithoides*	*L. lit*	AUM39040	no	yes
*Micracanthicus vandragti*	*M. van*	AUM54991	yes	no
*Neblinichthys yaravi*	*N. yar*	AUM36633	no	yes (*Neblinichthys echinasus* T06066)
*Panaque bathyphilus*	*P. bat*	AUM45504	yes (*Panaque cochliodon* 19170)	yes
*Panaque maccus*	*P. mac*	AUM22665	no	yes
*Paralithoxus bovallii*	*P. bov*	AUM67039, AUM67039, AUM67039, AUM67039, AUM67039	no	yes
*Peckoltia braueri*	*P. bra*	AUM48093	yes	yes
*Peckoltia ephippiata*	*P. eph*	ANSP197614, AUM42662, AUM65116, MCP48395, UF237091	no	no
*Peckoltia greedoi*	*P. gre*	ANSP197617, AUM21972, MCP21972, MNRJ42663	no	no
*Peckoltia lineola*	*P. lin*	AUM54033	no	yes
*Peckoltia n.sp.*	*P. n.s*	AUM21972	no	no
*Peckoltia sabaji*	*P. sab*	AUM35733, AUM38259, AUM39835, AUM48767	no	yes
*Peckoltia sp.*	*P. sp.*	MCBXXXXX	no	no
*Peckoltia vittata*	*P. vit*	AUM39313, AUM54314	no	yes
*Peckoltia wernekei*	*P. wer*	AUM39313	no	yes
*Peckoltichthys bachi*	*P. bac*	AUM45592, AUM66083	no	yes
*Pseudacanthicus leopardus*	*P. leo*	AUM35738	yes (*Pseudacanthicus* sp 64046)	yes
*Pseudancistrus barbatus*	*P. bar*	AUM38023	yes (*Pseudancistrus pectegenitor* 43192)	yes
*Pseudancistrus nigrescens*	*P. nig*	AUM44594, AUM45299	no	yes
*Pseudancistrus sidereus*	*P. sid*	AUM42168, AUM42180, AUM43443, AUM54310	no	yes
*Pseudolithoxus dumus*	*P. dum*	AUM39589, AUM42118	yes (*Pseudolithoxus tigris* 185263)	yes
*Pterygoplichthys gibbiceps*	*P. gib*	AUM41441	yes (*Pterygoplichthys multiradiatus* 47289)	yes
Loricariidae: Lithogeninae				
*Lithogenes villosus*	*L. vil*	AUM62909	no	yes
Loricariidae: Loricariinae				
*Crossoloricaria bahuaji*	*C. bah*	AUM51403	yes (*Crossoloricaria cephalaspis* 5106)	no
*Farlowella curtirostra*	*F. cur*	AUM46301	yes (*Farlowella oxyrryncha* 11509)	yes (*Farlowella acus*)
*Harttia platystoma*	*H. pla*	AUM35643, AUM38789	yes	yes (*Harttia loricariformis*)
*Hemiodontichthys acipenserinus*	*H. aci*	AUM44413, AUM51464	no	no
*Loricaria simillima*	*L. sim*	AUM57811	yes (*Loricaria prolixa* 34926)	yes (*Loricaria clavipinna*)
*Paraloricaria sp. (Dientes cortos)*	*P. sp.*	AUM39899	no	no
*Planiloricaria cryptodon*	*P. cry*	AUM57837	yes	no
*Pseudohemiodon sp.*	*P. sp.*	AUM41498, AUM27708, AUM39848	yes (*Pseudohemiodon lamina* 23059)	yes (*Pseudohemiodon laticeps*)
*Pseudoloricaria laeviuscula*	*P. lae*	AUM38888	yes	no
*Rineloricaria fallax*	*R. fal*	AUM47892	yes (*Rineloricaria maackii* 51110)	yes
*Rineloricaria stewarti*	*R. ste*	AUM44491	no	no
*Spatuloricaria puganensis*	*S. pug*	AUM45611, AUM46619	yes (*Spatuloricaria* sp 16145)	yes
*Sturisoma monopelte*	*S. mon*	AUM47971, AUM48752	yes (*Sturisoma barbatum* 42452)	yes (*Sturisoma* cf. *monopelte*)

### 2.2. Shape analysis

Landmarks were superimposed using a generalized least-squares Procrustes superimposition to remove non-shape-related information (orientation, translation, size) in the R package geomorph v. 4.0.1 [32,33]. To find the average shape for each species, individual superimposed landmarks were averaged using the aggregate function in the base package in R [34], and a multivariate analysis was performed through a principal component analysis (PCA). Landmark data were not corrected for phylogenetic relationships to determine trends and potential convergence in shape. The significant axes were found using the broken stick method in the R package PCDimension and principal component backtransformations were generated for significant axes to view the theoretical shape of the morphospace for both ventral and lateral views [35–39].

Phylomorphospaces for two well-supported phylogenies [21,22] were generated in the R package geomorph to explore the evolutionary trends in the loricariid body shape [32,33]. This method projects evolutionary relationships onto a shape space and estimates the ancestral shapes for the nodes to help visualize patterns in shape change across a phylogeny [40]. Phylogenies were downloaded from the respective supplemental materials and non-corresponding specimens were pruned from each tree in the R package ape [41]. Significant axes were found and backtransformations to visualize shape change were generated for each phylogeny. After pruning, the phylogeny generated by Lujan and others [21] had 49 corresponding species, covering four subfamilies, whereas the phylogeny generated by Roxo and others [22] had 30 corresponding species covering three subfamilies. Although the Lujan *et al*. [21] phylogeny had better coverage, the Roxo *et al*. [22] phylogeny is time-calibrated and represents a majority of species variation. The phylogenetic signal for each phylogeny was calculated using the Kmult method in geomorph, and the phylogenetic signal for each subfamily was found for the Lujan *et al*. [21] phylogeny [32,33].

Because the Lujan *et al*. [21] phylogeny covered a broader number of species, we assessed convergence and ecological correlation across the phylomorphospace. To determine if closely placed species converged in shape, we identified a group of species from different clades (*Corumbataia tocantinensis*, *Isorineloricaria spinosissima*, *Exastilithoxus* sp. and *Lithogenes villosus*) in the morphospace and performed convergence tests in convevol in R [42]. Using the function convnum, an ellipse was placed around the convergent taxa and the number of times a lineage crossed the ellipse was calculated. If a lineage (node to the tip of the phylogeny) crosses the ellipse, the respective taxa are suggested to be convergent [42]. To understand how morphological disparity changed over time, we calculated distance-based morphological disparity using code modified from Stanley [43] at 31 time points along the time-calibrated phylogeny [22]. Subfamilies were isolated and morphological disparity was calculated for these subsets. To calculate the disparity through time, ancestral shapes were reconstructed within a chronophylomorphospace, where the distance between the nodes was used to estimate ancestral disparity. Based on the overall disparity scores in the clade, an increase of divergence greater than 10 between two subsequent time points was determined to be a burst of divergence.

### 2.3. Modularity and ingression

We tested 10 *a priori* modularity model structures that ranged from fully integrated (one module) to highly parametrized (seven modules, figure 1). Hypotheses were based on an understanding of armoured catfish morphology and previous modular hypotheses for similar fishes (electronic supplementary material, table S1) [44]. To investigate patterns of modularity across the loricariid body, we used two approaches: a phylogenetically corrected evaluating modularity with maximum likelihood (EMMLiv2) and a covariance ratio (CR) analysis [45–47]. All further analyses used the Lujan *et al.* [21] phylogeny. EMMLiv2 uses maximum likelihood to test different modularity hypotheses and calculates the between- and within-module correlations for the best fit model to evaluate the degree of interrelatedness [47–49]. However, EMMLi has been found to favour parametrized models over smaller ones and does not explicitly test modularity hypotheses, so to support these findings, we used a CR method. Covariance ratio measures covariation between hypothesized modules [44,45]. Using the compare.CR function in geomorph, we tested for the best model and observed phylogenetically corrected patterns of modularity by using the phylo.modularity function (*α* = 0.5 with 1000 iterations) for the best supported model for all species and each subfamily [45]. An evolutionary rate ratio was used to calculate evolutionary rates among modules. Phylo.modularity calculates a ratio between multivariate rates, which are estimated for each module by replicating datasets along a phylogeny using a single rate Brownian motion model [44,50,51]. Lower values suggest greater modularity, where a CR = 1 suggests no modularity. Values above 1 mean covariance between modules exceed the covariance within the modules. To test for integration between modules, we ran phylogenetically corrected patterns of integration using phylo.integration for the best supported model for the whole family and each subfamily using an *α* = 0.5 with 1000 iterations [45]. Phylo.integration calculates the average pairwise partial least squares (PLS) under a Brownian motion model. rPLS closer to 0 suggests there is no integration between modules, whereas values closer to 1 suggest there is full integration between modules. We did not perform further adjustments for rPLS and CR tests, as concerns of elevated type I error using the phylo.modularity and phylo.integration tests have been rigorously tested by the geomorph package authors and found to be within appropriate rates [52–54].

We used the compare.multi.evol.rates function in geomorph to test for the evolutionary rates of each module for the best supported model for the family and each subfamily. To further understand the evolutionary rates of morphological change for each species and their ancestors, evolutionary rates were calculated for significant PCA axes (determined with the broken stick method mentioned above) using the function multirateBM in phytools. This penalized-likelihood model uses Brownian motion with a penalty term that is equal to the log-transformed probability density multiplied by an intermediate smoothing coefficient (*λ* = 1) [55,56]. Evolutionary rates were calculated across the Lujan *et al*. [21] phylogeny for the total shape and each separate module to test for differences among modules.

## 3. Results

### 3.1. Shape is driven by phylogenetic relationships

**Figure 2.**
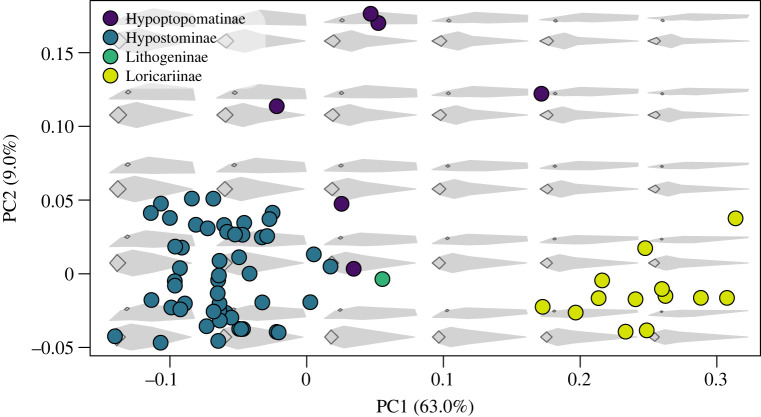
Body shape variation within loricariids. The morphospace of PC1 and PC2 represent approximately 70% of the body shape variation. Each point indicates the mean of a species, with colours matching the subfamilies denoted in the key. Backtransformed shapes (in grey with black outlined eyes and oral discs) portray shape variation throughout the morphospace.

The morphospace of loricariids showed clear separation between subfamilies, with the broken stick method finding two significant axes of shape variation (approx. 72.0% of variation) (figure 2). The first axis described approximately 63.0% of the variation in shape, where individuals on the negative end had thicker, deeper bodies, thicker caudal peduncles, larger oral discs and larger eyes. Individuals on the positive end of the first axis were slim and dorsoventrally compressed, with long, thin cauda peduncles, a smaller oral disc and smaller eyes. The second axis described approximately 9.0% of the overall variation in shape, which explained the placement of the eye. On the negative end, the eyes were placed more dorsally on the head, generally facing upwards, whereas the eyes on the negative end of the second axis were laterally placed, toward the middle of the head. The subfamily, Hypostominae grouped together toward the negative ends of both axes, whereas the Loricariinae grouped together toward the positive end of the first axis and negative end of the second axis. The Hypoptopomatinae were the most widespread subfamily across the morphospace but primarily grouped toward the positive end of the second axis. The subfamily, Lithogeninae was represented by one species which fell close to the intermediate shape (approx. *x* = 0.05 and *y* = 0) on the morphospace.

**Figure 3.**
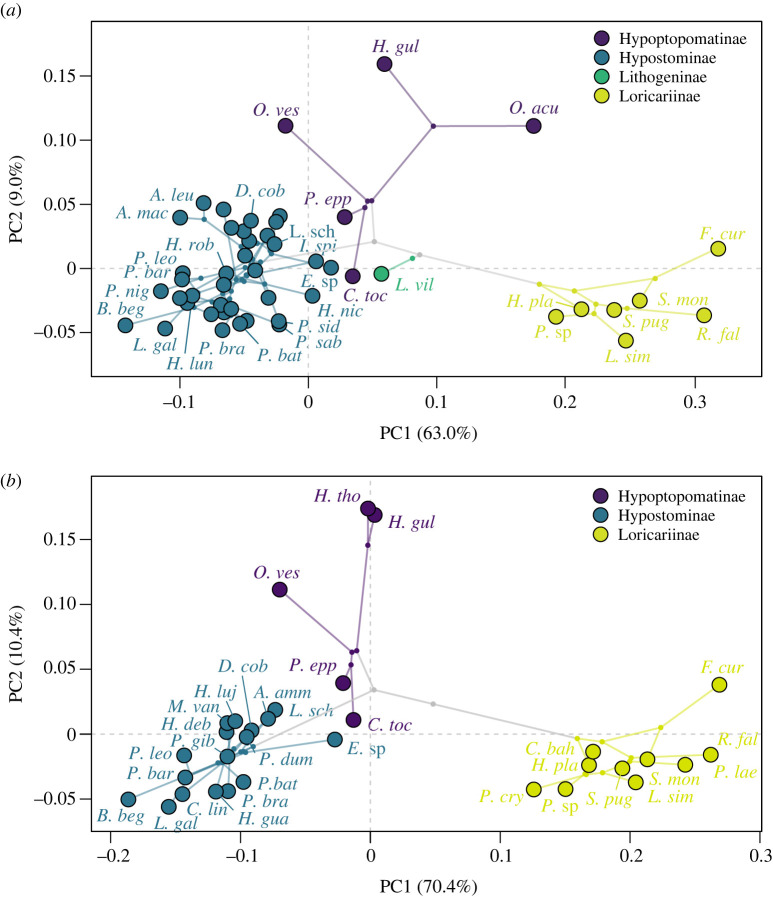
Phylomorphospaces of body shape across the loricariids. Phylogenetic relationships from (*a*) Lujan *et al*. [21] and (*b*) Roxo *et al*. [22] are projected onto the morphospace to demonstrate evolutionary relationships. Coloured points represent the mean of a species with ancestral nodes represented by small grey circles.

When the morphospace was trimmed to fit phylogenetic hypotheses, the broken stick methods found three significant axes for the Lujan *et al*. [21] phylogeny and two significant axes for the Roxo *et al*. [22] phylogeny (figure 3; electronic supplementary material, figures S1–S4). Shape variation across both phylomorphospaces were similar to the morphospace described above; however, the phylomorphospace based on the Lujan *et al*. [21] phylogeny showed additional changes in shape on the third axis, with the body shape being more compressed but thicker with smaller eyes on the dorsal part of the head and a wider oral disc on the negative end (electronic supplementary material, figure S3). On the positive end of the third axis, individuals were deeper bodied and thinner with larger more laterally placed eyes and a smaller oral disc (electronic supplementary material, figure S3). The observed phylogenetic signal for both phylogenies were significantly strong; the Lujan *et al*. [21] phylogeny had a *K* value of 1.1134 (*p* = 0.001) and the Roxo *et al*. [22] phylogeny had a *K* value of 1.1846 (*p* = 0.001). Within subfamilies, there were varying levels of phylogenetic signal for the Lujan *et al*. [21] phylogeny. The Hypostominae had a significant observed phylogenetic signal of *K* = 0.54 (*p* = 0.001), whereas Hypoptopomatinae and Loricariinae had insignificant *K* values of *K* = 0.96 (*p* = 0.1235) and *K* = 1.25 (*p* = 0.062), respectively.

Although subfamilies tend to cluster together, a few species from different clades seemed to converge in the intermediate shape space. The ellipsis covered an area of 3.797^e−4^ with all four species (*Corumbataia tocantinensis*, *Isorineloricaria spinosissima*, *Exastilithoxus* sp. and *Lithogenes villosus*) crossing the ellipsis (electronic supplementary material, figure S5). Convergent evolution was quantified using the C1, C2, C3 and C4 measures as described by Stayton [42] where the observed values are as follows: C1 = 0.737 (*p* = 0.00), C2 = 0.081 (*p* = 0.09), C3 = 0.348 (*p* = 0.00) and C4 = 0.035 (*p* = 0.00). To further explore convergent evolution between and within the subfamilies, we performed a PGLS for all specimens and for each subfamily.

### 3.2. Between subfamily divergence was fast

**Figure 4.**
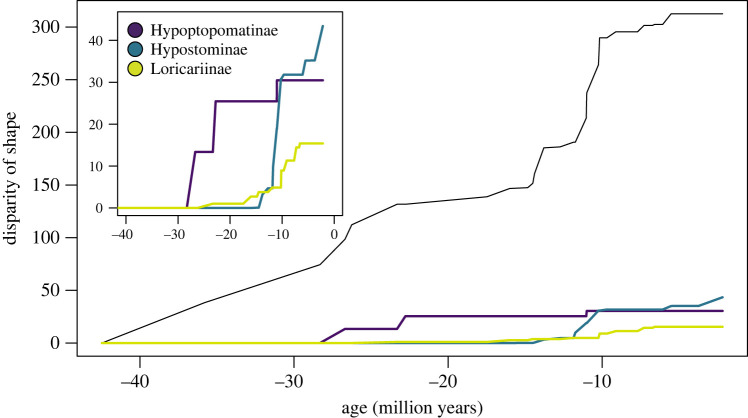
Disparity through time for loricariid family and separate subfamilies (inset) on the phylogenomic phylogeny (Roxo *et al*. [22]).

Overall, there were three major bursts of shape divergence across all species (figure 4, electronic supplementary material, table S2). The first happened primarily in the Oligocene, approximately 36–23 million years ago (Ma), when the subfamily Loricariinae diverged from the other subfamilies, Hypoptopomatinae and Hypostominae. This was followed by two bursts in disparity in the middle Miocene, approximately 14 and 11–10 Ma. Within the subfamilies, change in disparity varied in timing and speed. The Hypoptopomatinae was the earliest family to diverge approximately 28 Ma, with two quick bursts of disparity at approximately 27 (disparity = 13.37) and 23 Ma (disparity = 12.08) followed by a slow divergence for a total disparity of 30.46. This was followed by the Loricariinae which began to diverge approximately 23 Ma. Whereas the Hypoptopomatinae underwent fast changes in disparity, the Loricariinae experienced slow changes over time, steadily increasing to an overall disparity of 15.39. The Hypostominae was the latest to diverge approximately 14 Ma, experiencing a steady increase in diversity, followed by one major burst in disparity at about 10 Ma (disparity = 11.87) and a subsequent steady increase for a total disparity of 43.4. Overall the Loricariinae was the least disparate, whereas the Hypostominae was the most disparate subfamily.

### 3.3. Integration between modules may drive diversification

#### 3.3.1. Family level

**Figure 5.**
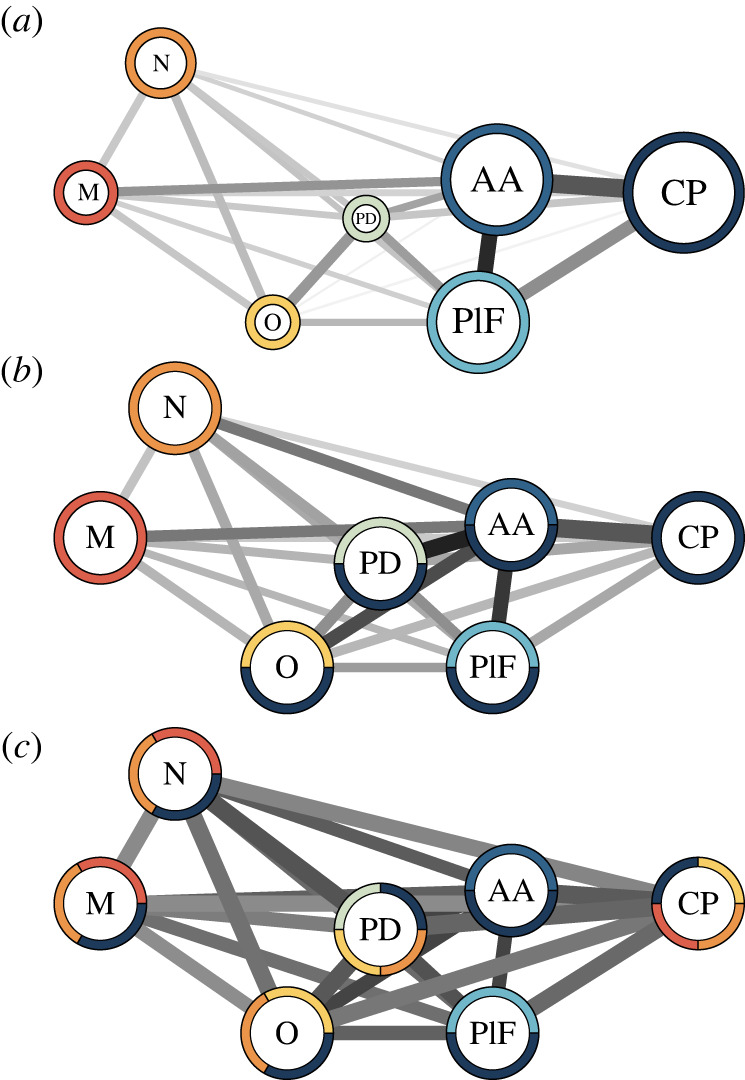
Modularity networks for the Loricariidae where lines represent between module integration. Networks are in approximant anatomical position where circles represent the module and colours represent high covariation (CR ≥ 1.00) or high integration (r-PLS ≥ 0.75) between modules. The size of coloured slices around which circles do not convey additional information. M represents the mouth (red), N the neurocranium (orange), O the opercula (yellow), PD the pectoral and dorsal fins (green), PIF the pelvic fins (light blue), AA the anal area (medium blue) and CP the caudal peduncle (dark blue). Where multiple colours suggest integration between the modules. (*a*) Phylogenetically corrected modules found by EMMLi where the size of circles indicated within modular integration. (*b*) Covariation ratios of modularity and (*c*) r-PLS integration tests are standardized to within modular integration, meaning the size of the circles does not convey additional information.

Phylogenetically corrected EMMLi analyses recovered the seven separate modules model as the best supported (electronic supplementary material, table S3). The pelvic fins (0.77), cloaca (0.84) and caudal peduncle (0.91) had strong within covariance, whereas the cloaca and caudal peduncle (0.81) had strong between covariance (figure 5*a*, electronic supplementary material, table S4). Because EMMLi tends to prefer the most parametrized model, we further tested model fit using a CR-based method which recovered the seven-module model as the best supported. Modularity tests recovered a slightly modular morphology (CR = 0.79, *p* = 0.001) (electronic supplementary material, figure S6). Pairwise CR suggests that the majority of the modules show some modularity; however, the caudal peduncle had high covariance with four separate modules, the opercula (CR = 1.04), the pectoral and dorsal fins (CR = 1.25), the pelvic fins (CR = 1.1) and the anal area (CR = 1.4). As these values are greater than 1, this suggests the covariance between modules exceeds covariance within each module, which suggests modules are highly integrated (electronic supplementary material, table S5). Integration tests for the best supported model found that there was some integration between modules as well (r-PLS = 0.73, *p* = 0.001). All pairwise r-PLS were significant (*p* = 0.001–0.002) and show varying amounts of integration between modules (electronic supplementary material, table S5). Based on arbitrary cut-offs, we see most modules have an intermediate amount of integration. We determined r-PLS ≥ 0.75 as high rates of integration between modules. Modules which show strong integration include: the mouth and neurocranium (r-PLS = 0.77, *p* = 0.001), the neurocranium and opercula (r-PLS = 0.81, *p* = 0.001), the neurocranium and pectoral/dorsal fin (r-PLS = 0.86, *p* = 0.001), the opercula and pectoral/dorsal fin (r-PLS = 0.81, *p* = 0.001) and the caudal peduncle with all modules (mouth at r-PLS = 0.76; neurocranium at r-PLS = 0.76; opercula at r-PLS = 0.81; pectoral/dorsal fin at r-PLS = 0.88; pelvic fins at r-PLS = 0.82; and the anal area at r-PLS = 0.86). The evolutionary rates for each module were similar to one another, with the exception of the caudal peduncle, which was approximately 5× higher than the other modules (electronic supplementary material, figure S7).

#### 3.3.2. Subfamily level

To examine modularity and integration patterns within subfamilies, we tested model fit using a CR-based method. The subfamily Hypostominae recovered a four-module model as the best supported (CR = 0.78, *p* = 0.001); where module 1 = mouth, module 2 = neurocranium and opercula, module 3 = pectoral, dorsal and pelvic fins and module 4 = anal area and caudal peduncle. Pairwise CR suggests most modules show some modularity, with the exception of the tail and midbody, which had high covariance (CR = 1.07) (electronic supplementary material, figure S7a, table S6). Integration tests for the best supported model found that there was some integration between modules (r-PLS = 0.797, *p* = 0.001). All pairwise r-PLS were significant (*p* = 0.001–0.006). Some modules show strong integration; the mouth with the tail region (rPLS = 0.76), the neurocranium/opercula with the tail (rPLS = 0.78), and the midbody with the tail (rPLS = 0.92) (electronic supplementary material, figure S7b). Evolutionary rates for each module were similar to one another, with the exception of the tail region which was approximately 3× higher than the other modules (electronic supplementary material, figure S8b).

The best supported model for the subfamily Hypoptopomatinae was a seven-module model (CR = 0.88, *p* = 0.001), the same model that was found to be best supported across the family. Pairwise CR suggest most modules show some modularity between one another, and many modules had very high covariance. The pectoral and dorsal fins had high covariance with the neurocranium (CR = 1.03) and the opercula (CR = 1.07), and the pelvic fins had high covariance with the anal area (CR = 1.05). Additionally, the caudal peduncle had high covariance with the pectoral and dorsal fins (CR = 1.13) and the anal area (CR = 1.21) (electronic supplementary material, figure S7c, table S7). Modules that had low covariance, suggesting stronger modularity between modules, were the mouth with the anal area (CR = 0.56) and the caudle peduncle (CR = 0.44). Integration tests for the best supported model were insignificant (r-PLS = 0.83, *p* = 0.21), with only one pairwise r-PLS value as significant (caudal peduncle with the anal area with a rPLS = 0.95, *p* = 0.047) (electronic supplementary material, figure S7d). The evolutionary rates for each module were similar to one another, with the exception of the caudal peduncle; however, these observed rates were not significant (*p* = 0.47) (electronic supplementary material, figure S8c).

For the subfamily Loricariinae, the best supported model was the seven-module model (CR = 0.88, *p* = 0.001), the same model that was found to be best supported for Hypoptopomatinae and the whole family. Pairwise CR suggests most modules show some modularity, yet some modules had high covariance. The pectoral and dorsal fins had high covariance with the neurocranium (CR = 1.05) and with the pelvic fins (CR = 1.07). The caudal peduncle had high covariance with the mouth (CR = 1.12), the opercula (CR = 1.07) and the anal area (CR = 1.13) (electronic supplementary material, figure S7e, table S8). Modules that had low covariance were the anal area with the pectoral and dorsal fins (CR = 0.52) and the pelvic fins (CR = 0.48). Integration tests for the best supported model found that there was some integration between modules (r-PLS = 0.808, *p* = 0.002). Some pairwise r-PLS were significant and show high integration between modules: mouth with the anal area (rPLS = 0.90, *p* = 0.018) and caudal peduncle (rPLS = 0.95, *p* = 0.008); the neurocranium with the pectoral and dorsal fins (rPLS = 0.95, *p* = 0.003) and pelvic fins (rPLS = 0.96, *p* = 0.003); the opercula with the pectoral and dorsal fins (rPLS = 0.82, *p* = 0.028), the pelvic fins (rPLS = 0.86, *p* = 0.037) and the caudal peduncle (rPLS = 0.90, *p* = 0.012); the pectoral and dorsal fins with the pelvic fins (rPLS = 0.92, *p* = 0.006); the pelvic fins with the caudal peduncle (rPLS = 0.81, *p* = 0.050); and the anal area with the caudle peduncle (rPLS = 0.88, *p* = 0.003) (electronic supplementary material, figure S7f). Evolutionary rates show the most variability between modules compared with all other subfamilies, including the total family (electronic supplementary material, figure S8d). The modules with the fastest evolutionary rates were the anal area, which was approximately 7× higher than other modules, and the caudal peduncle which was approximately 7.5× higher than other modules.

#### 3.3.3. Evolutionary rates

To further explore evolutionary rates across the loricariids, we calculated evolutionary rates using a penalized-likelihood model. There was no unique pattern to evolutionary rates for the total shape nor the rates for each module. For the total shape *Oxyropsis acutirostra*, *Otocinclus vestitus* and *Lithoxus lithoides* had the fastest evolutionary rates for PC1, PC2 and PC3, respectively. *Lithogenes villosus* had the slowest rates for both PC1 and PC2, yet *Harttia platystoma* had the slowest rates for PC3 (electronic supplementary material, figure S9, table S9). *Lithogenes villosus* had the slowest rates for each module, with few exceptions including the anal area for PC1 where *Lasiancistrus schomburgkii* had the slowest rates. The species with the highest rates varied for each module (electronic supplementary material, figure S10, table S9).

## 4. Discussion

The armoured catfish body shape strongly follows phylogenetic relationships across the family but shows different patterns of evolution within subfamilies. The loricariid subfamilies diverged early from one another (around 36–23 Ma) and show various levels of disparity within. Surprisingly, we found that the armoured catfish body is highly modularized, with varying degrees of integration between each module, which suggests that evolution of armoured catfish diversification is complex and morphological evolution is influenced by interactions within and between modules. Furthermore, the different patterns of evolutionary integration and modularity within the subfamilies may have allowed subfamilies to diversify along a single trajectory despite tight integration between modules.

Body shape is diverse within the Loricariidae, with shape ranging from dorsoventrally compressed with small eyes and a thin caudal peduncle to deep bodied with large eyes and a thick caudal peduncle. Additionally, we saw changes in the oral disc shape and size across the morphospace, with some species having wide and large oral discs whereas others had thin, small oral discs. The shape within the family is driven by phylogenetic relationships; however, within subfamilies there were mixed results. Although Hypoptopomatinae and Loricariinae had strong but insignificant phylogenetic signals, Hypostominae had a weak phylogenetic signal (*K* = 0.54, *p* = 0.001), suggesting that phylogeny may drive some shape variation, but not all. For example, a handful of species fell in the middle of the morphospace close to the root of the phylogeny (figure 3*a*). This suggests that *Corumbataia tocantinensis*, *Isorineloricaria spinosissima*, *Exastilithoxus* sp. and *Lithogenes villosus* retain the ancestral characteristics of the most common ancestor to the loricariids.

We found interesting patterns of divergence across the loricariids and within each subfamily (figure 4). Across the family, there were three quick bursts of morphological divergence, starting with the Loricariinae splitting from the other subfamilies around 36–23 Ma. During this time, there were many geological changes occurring in South America that may have contributed to the diversification and speciation of the loricariids. Around this time the central and northern Andes began to uplift and the sub-Andean trunk river flowed south to north into the Atlantic Ocean [57]. Although there are some loricariines at high elevation, loricariines tend to be more diverse in the lowlands, and the early orogeny of the Andes may have allowed for greater isolation of foreland basins. Interestingly, there was a second and third burst of morphological disparity around the middle Miocene (approx. 14–10 Ma). This time is referred to as the middle Miocene disruption, which is associated with global cooling and aquatic extinctions, yet South America experienced even more drastic changes with the orogeny of the Andes and the formation of the Amazonian river system which flows west to east. Many groups of fishes, other than the loricariids, have undergone similar diversification patterns, which are documented in many marine fishes [58,59]. Increased extinction rates in addition to the formation of new habitats may have led to the further diversification of the armoured catfishes. For example, the rise of the Andes allowed for diversification of high montane taxa in sub-basins as well as differentiation between species in cis- versus trans-Andean basins (cis- refers to areas east and south of the Andes and trans- for areas west and north). The developing Amazon River began to capture other river systems, as can be seen today with the Casiquiare River (which drains much of the upper Orinoco into the Amazon) and the Rupununi Portal (which seasonally connects the Amazon and Essequibo rivers). These major shifts in river basins allowed for isolation of formerly connected habitats as well as movement of Amazonian fauna into other river systems, which have continued to accelerate speciation in Neotropical fishes [57,60,61].

Within the subfamilies, different patterns of morphological disparity emerged. The subfamily Hypoptopomatinae began to diversify around 28 Ma with two bursts in shape resulting in a moderate variation in body shape. Admittedly, this family is represented by few species in our dataset, so the patterns in disparity may be exaggerated. The least disparate subfamily was the Loricariinae, which began to diverge and steadily diversify around 23 Ma. This subfamily occupied a small region of the morphospace, which suggests shape evolves more gradually than in other subfamilies. This may be because of limitations enforced by the extreme dorsoventral flattening in loricariines (figures 2 and 3). The Hypostominae experienced a steady increase in disparity, starting around 14 Ma, resulting in the subfamily becoming the most disparate group of the loricariids. The hypostomine body form seems less constrained than that of hypoptopomatines or loricariines. Hypostomines have a broader range of size disparity in addition to shape disparity when compared with all other loricariid subfamilies. Hypostominae includes species nearly as elongate as loricariines (*Isorineloricaria* was named because of its similarity in form to loricariines [62,63]); as well as species approaching the small sizes of some hypoptopomatines [61] and some among the largest of loricariids [64]. Biogeography probably plays a role in diversity as well. Hypoptopomatines are more diverse in species and morphology in the shorter Atlantic drainages of Brazil, and these smaller river systems do not provide the breadth of habitats available elsewhere where hypostomines are dominant [65,66]. Hypostomines and loricariines share similar continental ranges, but hypostomines are diverse in both uplands and lowlands whereas loricariine diversity in uplands is lower (Armbruster, personal observation).

Independently evolving modules may allow for greater morphological diversification, and we found that loricariids are highly modularized, with varying degrees of integration between each module (figure 5). Both likelihood and covariation ratio models returned a seven-module system across the family of armoured catfishes, giving the fishes many areas to adapt with some degree of independence from one another; however, varying degrees of integration between the modules means that changes within one module will probably lead to cascading changes across the body. Specifically, morphological changes in the caudal peduncle are highly integrated with the rest of the body. This means if shape changes occur in the caudal peduncle, for example if the caudal peduncle becomes thinner, the rest of the body will experience morphological changes to some degree. We also found the neurocranium, opercula and pectoral/dorsal fins had a strong degree of integration, which could explain the relatively small and weak fins and heads of loricariines versus the broader, deeper heads and larger fins of hypostomines. The tight integration between the neurocranium and pectoral fins is not surprising due to the fusion of the neurocranium and pectoral girdle which limits movement in the head; however, we are unable to test these specific functional implications as our dataset does not include specific landmarks that may explain these functional implications. Additionally, integration and modularity are not all-or-nothing concepts, this suggests that each module is separate from one another to some degree, but not completely independent of one another [18]. Our hypothesis that different jaw morphologies may be found in similar body types (i.e. jaw module swapping) may not be supported by these findings as when one module changes, it will influence the other modules. However, the interplay between modularity and integration within the loricariid body may attribute to the high degree of diversity that is seen within these fishes.

In addition to patterns of modularity and integration at the family level, we found slight differences between modularity and integration within the three main subfamilies (Lithogeninae was removed from the dataset due to low species sampling, *n* = 1 (3 total species)), which may explain why subfamilies occupy their own area of the morphospace. Both the Hypoptopomatinae and the Loricariinae were highly modular yet show differences in what modules are more covariant with one another. The Hypoptopomatinae show high covariation of the pectoral, dorsal and pelvic fins with the anal area, whereas the Loricariinae had little covariation between those modules. Conversely, the Loricariinae have high covariation between the mouth, the anal area and the caudle peduncle, where the Hypoptopomatinae had little covariation of the modules. Interestingly, the Hypostominae has less modularity than the other subfamilies, with a four-module system. Our data suggest that the mouth acts as a module separate from the neurocranium and opercula. Furthermore, the pectoral, dorsal and pelvic fins act as a module and the anal area and caudal peduncle acts as the final module. Although there were high amounts of integration between modules, this separation of mouth from other parts of body may allow for changes to the rest of the body while retaining similar feeding modes. These slight changes in modularity and integration patterns may have allowed for different interactions of parts to increase diversity along an independent trajectory for each subfamily. This has been demonstrated in phyllostomid bats where Hedrick *et al*. [17] showed that cranial morphology evolved from other bats by following a single morphological axis that describes the relative length of the rostrum. By developing various rostrum lengths, these bats were able to occupy a larger range of diets, suggesting they followed a line of least evolutionary resistance. Perhaps each subfamily of loricariid has evolved along a line of least evolutionary resistance to adapt to specific habitats.

When confronted with the dizzying array of morphological diversity proscribed by groups of organisms like loricariids, it is difficult to understand how such diversity has evolved. Loricariids are especially problematic as most eat an unidentifiable mixture of organic compounds and biofilm that make dietary description difficult. Stable isotopic studies have not shown great diversity in what loricariids assimilate from the environment, making the array of forms within the family particularly confounding [67,68]. Although ecological reasons for diversity of form are still elusive, the great number of morphological modules found in this study demonstrate a proximate reason such morphological diversity has formed. Those morphological modules have some evolutionary independence from one another to evolve separately and varying degrees of integration between modules means that evolutionary pressures to change one part of the body will have concomitant changes across the body. This tight interplay between many integrated modules allows for the morphological diversification observed and explains patterns such as that demonstrated by *Peckoltia lujani* and *P. wernekei*, which differ in jaw shape (long and nearly straight dentaries versus short, angled dentaries), tooth number and size (many small versus few larger) and body shape (elongate and narrow versus short and stout) despite having little genetic differentiation [69]. Phylogeny was found to be a driving factor for diversification of the family, but not within subfamilies, meaning that convergence probably plays a major role in the evolution of form and integrated modules may further prompt convergence of morphologies. Our study was necessarily limited by the scope of phylogenies available, and as knowledge proceeds, a study such as this will be able to capture more of the morphological variation present within the family.

## Data Availability

Data and relevant code for this research work are stored in GitHub: https://github.com/corinthiablack/ArmoredCatfish-Diversification; and have been archived within the Zenodo repository: https://doi.org/10.5281/zenodo.7296585 [70]. The data are provided in the electronic supplementary material [71].
